# Wearable Cameras Reveal Large Intra-Individual Variability in Timing of Eating among Young Adults

**DOI:** 10.3390/nu14204349

**Published:** 2022-10-17

**Authors:** Leanne Wang, Virginia Chan, Margaret Allman-Farinelli, Alyse Davies, Lyndal Wellard-Cole, Anna Rangan

**Affiliations:** 1 Discipline of Nutrition and Dietetics, Susan Wakil School of Nursing and Midwifery, Faculty of Medicine and Health, The University of Sydney, Sydney, NSW 2006, Australia; 2 Charles Perkins Centre, The University of Sydney, Sydney, NSW 2006, Australia; 3 Cancer Prevention and Advocacy Division, Cancer Council NSW, Sydney, NSW 2011, Australia

**Keywords:** meal timing, variability, wearable camera, young adults, nutrition, eating occasion, food consumption, eating window, intra-individual variability

## Abstract

Studies have shown that young adults follow less structured eating patterns compared with older cohorts. This may have implications for dietary assessment methods which rely on memory and structured meal patterns. Our aim was to describe the intra-individual variation of eating times in young adults aged 18–30 years. Participants (*n* = 41) wore an Autographer camera that captured first-person perspective images every 30 s for three consecutive days. All images were timestamped and those showing food consumption were used to extract data such as the timing of the first and last eating occasions (EOs), number of EOs per day, and length of eating window. Intra-individual variability was calculated from these data using composite phase deviation (CPD) and coefficient of variation (CV). The number of individuals with high or very high variability was 28 and 18 for timing of first and last EOs, respectively (CPD > 1.70), and 27 and 17 for number of EOs and eating window, respectively (CV > 20%). In this sample of young adults, the lack of regularity in eating patterns should be considered when selecting a dietary assessment method.

## 1. Introduction

Previous studies have shown that young adults eat at later times throughout the day, follow less structured eating patterns, and have a higher variability in energy intake when compared with older cohorts [1,2,3,4,5]. It is unclear whether this has an impact on dietary quality and health due to the heterogenous nature of the studies exploring this relationship in terms of methodology used and outcomes investigated as reported in previous reviews [6,7,8,9]. Common outcomes include meal frequency (number of meals per day), breakfast consumption or lack thereof [3,10,11,12,13,14], late-night eating [14,15,16,17,18,19], time-restricted eating such as intermittent fasting [8,20], and meal timing relative to sleep timing [21,22,23]. In recent years, there has been a greater interest in the intra-individual day-to-day variability of meal timing and its association with health outcomes [4,21,24,25,26,27,28,29,30,31,32,33]. 

Methods of data collection in meal timing variability studies are usually subjective. There is a heavy reliance on self-report and human recall such as dietary recalls [4,25,31,32,33,34], questionnaires [24,28,35,36,37,38], or user-initiated food image capture [5,21,27,30]. This, combined with the availability of feedback from these data collection methods, may influence or interfere with subjects’ food choices and behaviors [5]. Technologies such as wearable cameras present an opportunity to objectively monitor human food and beverage consumption. They can automatically capture time-stamped images of all activities throughout the day, including eating occasions, without requiring user input and can be analyzed for daily eating patterns [5,39,40].

In this study, we examine young adults’ eating patterns via time-stamped food images obtained from wearable cameras. Specifically, for our first aim, we sought to describe the intra-individual variation of eating times in young adults using four different metrics and categorize them as low, moderate, high, or very high variability. Our second aim was to investigate whether there was an association between the four metrics used to measure intra-individual variability and specific demographics such as gender, age, body mass index (BMI), and socioeconomic status (SES), as well as total energy intake across the duration of data collection.

## 2. Materials and Methods

### 2.1. Data Collection

This paper used data from the sub-study (*n* = 133) [41] of a larger cross-sectional MYMeals project (*n* = 1001) [42]. Participants in the sub-study were young adults (18–30 years) who wore an Autographer camera for three consecutive days. The wearable camera was worn on a lanyard around the neck and automatically captured images from a first-person perspective every 30 s. Participants were instructed to wear the camera for all waking hours over the three days while maintaining their usual daily activities. They were permitted to temporarily halt image capture or remove the camera when privacy was needed [43,44]. On the same three days, they also recorded food and beverage intake using a researcher-designed smartphone app named EaT and Track (The University of Sydney, Sydney, Australia) [45] and completed daily 24-h dietary recall interviews with research dietitians via the Automated Self-Administered 24-h recall Australia program (Deakin University, Melbourne, Australia) [46]. Recruitment methods have previously been described in the MYMeals study protocol [47]. In brief, participants had to be between the ages of 18–30 inclusive, consume foods or beverages prepared outside the home at least once a week, own a smartphone, and read and write English. Participants who were pregnant, lactating or had ever had an eating disorder were excluded. Participants completed a basic demographic questionnaire, providing information such as gender (male, female or prefer not to say), education status (primary school or less, secondary school, trade qualification/apprenticeship/diploma or university degree), employment status (full-time study, full-time work, part-time study/work, not working or studying) and residential postcode. Residential postcode was used to determine relative socio-economic advantage and disadvantage ranking within Australia using the Socio-Economic Indexes for Areas 2016 (high; top five deciles or low; bottom five deciles) [48]. Participants’ weight (kg) and height (cm) were self-reported and used to calculate BMI (underweight < 18.5, healthy weight 18.5–24.9, overweight 25–29.9, obese ≥ 30 kg/m^2^). Using self-reported weight and height to calculate BMI has been found to be sufficiently accurate [49]. Deidentified camera images were stored in the university’s research data store and demographic and anthropometric information were hosted and stored in the Research Electronic Data Capture (REDCap) data management system (Vanderbilt University, Nashville, TN, USA) [50]. This sub-study was approved by the Institutional Human Research Ethics Committee (2016/546) on the 15 July 2016.

All images captured by the Autographer camera were coded by an Accredited Practising Dietitian for the presence or absence of food and beverages and then matched with their 24-h recall data. This involved matching the time and date stamp of the wearable camera images with the self-reported times of the 24-h recall and annotating foods and beverages reported in the 24-h recall as: (i) reported by both methods, (ii) not reported in the 24-h recall, or (iii) not identified by the wearable camera (i.e., the camera may have been turned off). For entries labelled as not reported in the 24-h recall or not identified by the wearable camera, the omitted episode and associated food and beverage items were tabulated in Microsoft Excel. Two researchers checked all matching of data from the three sources (v.c. and a.d.) [40]. 

### 2.2. Inclusion Criteria

The 24-h recall was considered the ground truth method and foods that were reported in the 24-h recall but not identified by the camera were labelled as missing. If foods from an entire meal or snack were missing from the images, the eating occasion (EO), i.e., main meal or snack, would be labelled as missing. To ensure the inclusion of only high quality data, participants with no missing main meals and no more than three missing snacks over the course of the three days of data collection were included for analysis in this paper. Data from the EaT app and images of beverages were not used, as it was difficult to differentiate between nutritive and non-nutritive beverages, e.g., images of opaque drinking utensils.

### 2.3. Data Analysis

The times of EOs were collated using the time and date stamps available from the camera images. An EO was defined as the consumption of any food. In cases where there was more than one image captured of an EO, the timestamp of only the first image (i.e., the one with the earliest time) was used to represent its time of consumption. EO labels such as main meals or snacks were not used and were only labelled by time of day. Any EO that was recorded ≤ 15 min of a previous event was combined to form one EO as per previous studies [4,5]. Each day was defined as the 24-h period from 12:00 am to 11:59 pm on the same calendar day. No drinking occasions were included.

Eating pattern metrics such as the total number of EOs per day (after combining events within 15 min of each other), the clock time of the first and last EO of each day, the daily eating window, and the daily energy intake were extracted for all days. The daily eating window was defined as the duration between the start time of the first EO to the start time of the last EO of the same day. These metrics were stratified by weekdays and weekends and also compared between participants with healthy and overweight/obese BMIs. 

#### 2.3.1. Meal Timing Variability Metrics

Four metrics were applied to assess the stability of meal timing across the three days of data collection: Composite Phase Deviation of the first EO (CPD First), Composite Phase Deviation of the last EO (CPD Last), coefficient of variation of the daily number of EOs (CV No. of EOs), and coefficient of variation of the daily eating window (CV Eating Window).

The first two metrics both used Composite Phase Deviation (CPD) to assess the day-to-day stability of meal timing [21] for the first and last EO of each calendar day. CPD combines two components: (i) regularity, i.e., how different the timing of a particular meal is from the same meal of the previous day, and (ii) alignment, i.e., how different the timing of a particular meal is from the average time of that meal over the number of days of data collection [51]. The greater the CPD score, the greater the deviation in hours from a perfectly regular pattern of meal timing, i.e., the same time every day. CPD was originally developed to assess the stability of sleep timing but has recently been applied to social and eating events [21,52]. This is the formula for CPD:

ΔRegularity_i_(ΔDD_i_) = Meal timing_i–1_ − Meal timing_i_

ΔAlignment_i_(ΔAT_i_) = Average meal timing − Meal timing_i_

CPD_i_ = √(ΔDD_i_^2^ + ΔAT_i_^2^)

CPD = 1/N(∑^N^_i=1_ CPD_i_)

Variables: DD stands for “day-to-day”, AT stands for “average timing”, i is any given day, and N is the total number of days.

The third and fourth metrics both used coefficient of variation (CV) to evaluate intra-individual variability. The formula for CV is: (standard deviation/mean) × 100. One measured the intra-individual day-to-day variability of the total number of EOs per day and the other measured the intra-individual day-to-day variability of the daily eating window. 

The cut-offs used to categorize CPD and CV scores as low, moderate, high, and very high variability were based on hours or number of EOs (Table 1). For example, participants with low variability in CPD of the first EO varied in meal timing by less than two hours over the three days of data collection. An example of this is the consumption of the first meal or snack at 9:00 a.m. on the first day, 10:00 a.m. on the second day, and 10:45 a.m. on the third day. Participants with high variability in CV number of EOs differed by more than three EOs over the three days.

#### 2.3.2. Statistics

Statistical analyses were conducted using SPSS software, v27.0 for Windows (IBM, Armonk, NY, USA). To test for differences in intra-individual meal timing variability metrics between demographic variables and eating pattern metrics between BMI categories (reduced to two categories—healthy weight versus overweight/obese), the Mann-Whitney U test was used. Spearman rank-order correlation coefficients were used to identify associations between energy intake and intra-individual variability metrics. A *p*-value of ≤0.05 was considered statistically significant. 

## 3. Results

The MYMeals sub-study recruited 133 participants, of which 41 met the inclusion criteria for this paper. Participant characteristics including age, BMI, SES, education level, and employment status can be found in Table 2. A total of 577 EOs across 123 days were included for analysis. Of the 123 days, 72% (*n* = 88) were weekdays and 28% (*n* = 35) were weekend days. 

The mean and range of eating patterns and intra-individual variability metrics are shown in Table 3. The average time of first and last EO were 10:18 a.m. and 8:06 p.m., respectively. The average number of EOs per day was 4.7, the average length eating window duration was 9.9 h, and the average daily energy intake was 8478 kJ. The intra-individual variability of the first EO was greater than the last EO as measured by CPD (2.9 versus 1.8), i.e., the time at which participants consumed their last EO over the three days was more consistent compared with the first EO. 

Table 4 compares the eating pattern metrics of participants with a healthy versus an overweight/obese BMI. Although the window of time in which participants consumed food over the course of the day and the daily energy intake were slightly higher for people with overweight/obese BMIs, this was not statistically significant. All other eating pattern metrics also showed no statistically significant differences between BMI groups.

A comparison of the mean and range of eating patterns and intra-individual variability metrics between weekdays and weekends is shown in Table S1. Whilst the mean eating window was larger on weekdays than weekends (10.1 h versus 9 h), the mean daily energy intake was greater on weekends than weekdays (8875 kJ versus 8320 kJ). 

**Figure 1:**
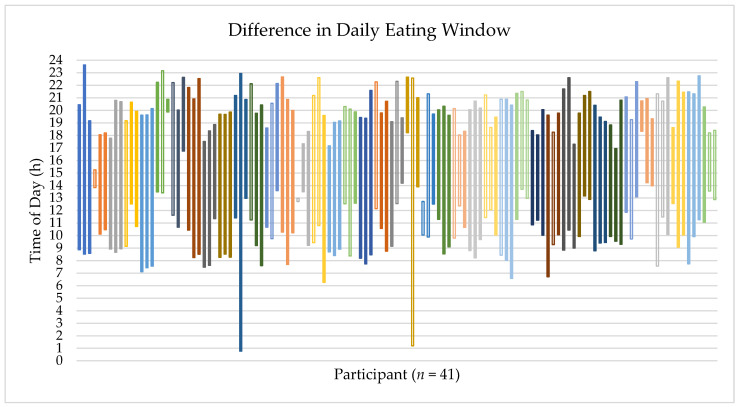
Individual differences in eating window (time from the first eating occasion of the day until the last eating occasion of the day). Each color represents a separate participant. Each column represents one day. Solid columns represent weekdays and unfilled columns represent weekend days.

Figure 1 is a visual representation of the three eating windows for each participant. The shortest observed eating window was 15 min, and the longest was 22 h 11 min. Ninety five percent of participants (*n* = 39) had at least one day out of the three days with an eating window of < 12 h. Fifty nine percent of participants (*n* = 24) had an eating window of < 12 h for all three days of data collection.

Table 5 compares the intra-individual variability metrics for each of the demographic variables studied. There were no significant associations for age, gender, and BMI, but individuals from a high SES (top five deciles) had a significantly greater variability in the timing of the first EO compared with individuals from a low SES (bottom five deciles) (CPD First 3.8 versus 1.9, *p* = 0.047).

**Figure 2:**
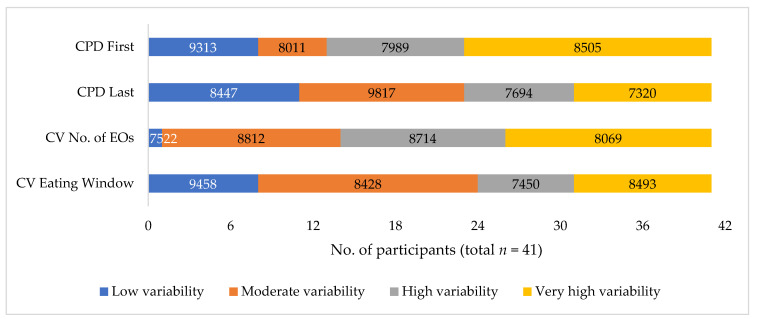
Stacked bar chart showing the number of participants that fall into the low, moderate, high, and very high intra-individual variability categories for each metric. Numbers within each category represent the average daily energy intake (kJ) for participants in that category. CPD First: Composite Phase Deviation of the first eating occasion for each participant over the three days of data collection. CPD Last: Composite Phase Deviation of the last eating occasion for each participant over the three days of data collection. CV No. of EOs: Coefficient of variation for the total daily number of eating occasions for each participant over the three days of data collection. CV Eating Window: Coefficient of variation for the daily eating window for each participant over the three days of data collection.

Figure 2 shows the distribution of participants in the low, moderate, high, and very high variability categories for each meal timing stability metric. For the first EO (CPD First), 28 out of 41 participants had high to very high variability. This is similar (*n* = 27) to the variability for the number of EOs (CV No. of EOs). In contrast, variability was lower for the last EO (CPD Last) and the daily eating window (CV Eating Window) with high to very high variability in 18 and 17 participants, respectively. There were no participants who fell into the low category for all four metrics simultaneously, whereas six participants had all four metrics in the high or very high variability category.

Total energy intake across three days was not significantly associated with any of the metrics used to measure day-to-day variability of meal timing (Table S2). The average daily energy intake (kJ) for participants in the low, moderate, high, and very high variability categories for each metric is shown in Figure 2. 

## 4. Discussion

Overall, our results showed that the timing of the first EO and the daily number of EOs were highly variable from day-to-day. It was common for the timing of the first EO to vary by more than three hours across days and for the number of EOs per day to vary by more than two EOs. In contrast, the timing of the last EO and daily eating window were relatively more stable with more participants in the low to moderate variability groups. Most of the participants in these groups had less than three hours of variation for the timing of the last EO and less than four hours of variation for the daily eating window. These results add to previous studies which have found that young adults tend to eat more frequently and erratically throughout waking hours [4,5].

### 4.1. Comparing Our Methods and Results with Previous Studies

Studies that measure the intra-individual variability of eating timing use subjective methods of data collection and have inconsistent definitions for meal time variability or regularity. These methods include using metrics such as CPD, CV, SD, and mean meal shift [21,25,27,30,31,32,33], calculating the proportion of daily energy intake per hourly bin [4,5,26,27,29,30,32,34], using self-reported questionnaires [24,28,36,37,38], measuring eating jetlag between weekends and weekdays [28,31], and assessing meal frequency [53,54,55]. The metrics that we developed for our paper (CPD and CV) were adapted from methods used by McHill et al. [21] and Popp et al. [30] but adjusted for three days of meal timing data. Previously, epidemiology papers have established cut-offs for CPD and CV to categorize scores as low, moderate, or high variability for sleep timing [56,57,58]. However, these cut-offs were based on levels of sleep variability that were associated with increased health risks and were not appropriate for categorizing meal timing variability [58]. It is important to establish standardized cut-offs for eating patterns to effectively compare results between dietary studies. 

McHill et al. evaluated intra-individual variability of food intake in healthy young adults across two timescales—a daily timescale (seven days) using CPD and a monthly timescale (seven days per month for three months) using intra-class correlation. They found that the CPD score for the last caloric event was the highest (i.e., least consistent across days) relative to the first caloric event and caloric midpoint. This contrasts with our results, where we showed that CPD Last was lower than CPD First. While no association was seen between BMI and meal timing variability in our study, McHill et al. found that non-lean individuals had a higher stability in meal timing when measured between months [21]. Popp et al. measured intra-individual variability in a population of adults who were overweight or obese using CV No. of EOs and the CV of the timing of the first and last EO over two or more days. Their results indicated that, compared to weekdays, the timing of the first EO was later but more consistent and that there were fewer EOs and a shorter eating window on weekends. This is consistent with our results which also saw a later first EO and shorter eating window on weekends but a similar number of EOs across weekdays and weekends. Popp et al. also found no significant differences between genders for all metrics, aligning with our findings [30]. 

### 4.2. Implications of Meal Timing Variability on Dietary Quality and Health

Most epidemiological findings appear to suggest that following eating patterns with a large variability in meal timing has negative implications on dietary quality and/or cardiometabolic health [27,28,29,30,31,32,33,34,37,38,54]. Data collected in the 1994–2004 National Health and Nutrition Examination Survey (NHANES) and 2005–2007 German National Nutrition Survey II (NVS II) showed that a regular meal pattern with greater energy intake earlier in the day scores higher on the Healthy Eating Index [29,34]. This provides physiological benefits such as a reduced prevalence of metabolic syndrome, reduced waist circumference, improved HDL cholesterol, and reduced γ-glutamyl transferase concentrations in Swedish men and women aged 60 years or above [37]. A prospective study also showed an association between irregular eating at 16 years of age and the development of metabolic syndrome at the age of 43 [38], although this was explained by concurrent unhealthy lifestyles such as smoking, alcohol, and low levels of physical activity.

Makarem et al., Meth et al., and Zhao et al. used SD to measure intra-individual variability in a range of populations including women aged 20–64 years with cardiometabolic risk factors [31,33], 70-year-old men [25], and adults with overweight or obese BMIs [27]. All three studies found that a high day-to-day variability was associated with adverse effects on health such as an increased blood pressure, worse glycemic control [31], elevated high-sensitivity C-reactive protein [33], higher fatal cancer risk [25], and increased adiposity [27,31]. These results are also supported by Fleischer et al. for healthy adults without obesity [32]. Eating jetlag, defined as weekday-weekend differences in meal timing, has been measured in two studies [28,31]. Zerón-Rugerio et al. showed that an eating jetlag of 3.5 h or more was significantly associated with an increased BMI in young adults aged 18–25 years [28], and this was supported by Makarem et al. who found similar associations both cross-sectionally and longitudinally in women with cardiometabolic risk factors [31]. 

Given the negative health effects of eating irregularly, three randomized crossover trials have examined the effects of a consistent versus an inconsistent number of meals per day on the cardiometabolic health of lean [54,59] and overweight or obese women [53]. A regular meal pattern where participants were provided six meals a day for 14 days had a beneficial impact on peak insulin and fasting total and LDL-cholesterol levels when compared to an irregular meal pattern varying from three to nine meals a day for the same duration of time [53,54]. Although only one study out of all of the above looked specifically at meal timing variability in young adults [28] overall, they all show that following eating patterns with large variability has negative implications with regard to various health outcomes.

In contrast, McHill et al. found that young adults who are overweight or obese have a higher stability in meal timing across months [21], which contradicts the findings of previous studies [28,34]. The authors explained that this may have been due to the lack of consistency in the definition of meal timing stability [21]. Two large studies found no statistically significant association between meal time regularity and cardiometabolic outcomes such as metabolic syndrome [36] and BMI [24]. One of these studies was conducted in a sample of 5337 Korean men aged 30 years and above [36], whereas the other was conducted in 1175 healthy UK adults aged 19–64 years [24].

The mechanisms behind the impact of meal timing variability on cardiometabolic health are yet to be fully understood due to the limited number of observational and intervention studies in humans [60]. However, it has been hypothesized that the timing and regularity of meals play an important role in entraining circadian rhythms [31]. Although the master clock in the brain is regulated by exposure to light, meal timing is a strong cue for peripheral clocks [61,62], which are present in nearly all organ systems [63,64]. These systems are involved in nutritionally-related metabolic processes such as glycolysis and gluconeogenesis, cholesterol and lipid metabolism, oxidative phosphorylation and detoxification [64,65]. Studies have shown that an irregular eating pattern, defined as an inconsistent number of EOs from day to day [59] or a change in the time of meal consumption [66], could lead to circadian misalignment [66], and has been linked with lower energy expenditure, greater hunger ratings, and lower fullness rating, resulting in a positive energy balance which potentially increases the risk of developing obesity and cardiometabolic diseases [59]. Animal studies have exposed genes that link circadian rhythms with metabolic regulation such as the circadian locomotor output cycles kaput gene in mutant mice. Mice lacking this gene were hyperphagic, obese, and rapidly developed metabolic syndrome [67]. The relationship between food intake and circadian rhythms is reciprocal—circadian rhythms drive changes in metabolic pathways and changes in metabolic pathways alter molecular components of circadian rhythms [68,69].

Having a high variability in eating patterns also has implications for dietary assessment. Traditional dietary assessment methods that rely on human memory to initiate the recording of food intake or recall foods consumed such as food diaries and 24-h recalls are high burden and subject to memory bias [70,71]. Important information such as foods consumed at breakfast may be omitted as its timing may vary considerably from day-to-day. Increasing the frequency of data collection throughout the day such as progressive 24-h recalls [72] or collecting dietary information near real-time may be warranted for this cohort. This can be achieved with the use of time-triggered ecological momentary assessment (EMA) to capture food intake and contextual data at pre-determined times personalized to subjects’ usual pattern of food intake [73,74]. Although not yet commercially available nor feasible for use in practice settings [75], wearable sensors may also be useful for capturing real-time eating behavior [76]. Sensors embedded in smartwatches can be used to detect eating-related behaviors such as hand-to-mouth movements to deliver timely prompts via a connected device such as a smartphone to remind users to record their dietary intake, minimizing inaccurate dietary recalls because of memory decay [77,78].

### 4.3. Strengths and Limitations

A key strength of our study is the use of wearable cameras. To the best of our knowledge, our paper is the first to use automated wearable cameras to objectively measure the intra-individual variability of meal timing in young adults. Previous studies have either used methods of data collection that are subjective [4,24,28,31,34,35,55] and require user-initiation [21,30] or examined meal timing in terms of when meals and snacks are consumed rather than its consistency or variability across days [79]. 

Our study had several limitations, the most notable being the small sample size. Our results are likely not generalizable to the larger population, and one example of this is the disparity between the proportion of our sample who had overweight and obese BMIs and the young adult Australian population (32% versus 46%) [80]. Another limitation is the exclusion of beverages from our analysis despite beverages contributing up to one quarter of total daily energy intake [81,82]. Including nutritive beverages may change the intra-individual variability of consumption timing. The data that we collected may not be representative of participants’ usual intake, as they only wore the camera for three days, may have altered their eating behavior on the days the camera was worn, or chosen to wear the camera on days that were more convenient such as at home or away from social settings. Week-long cross-sectional studies are likely needed to capture habitual eating behaviors [21]. 

## 5. Conclusions

Our results contribute to the literature on the timing of eating patterns in young adults and highlight the urgent need to develop more objective methods of dietary assessment that rely less on human recall. Methods that are based on data capture around the traditional structure of meal times are not appropriate for this cohort. However, to date, some of our innovations in the use of technology via apps and websites for dietary data collection have still maintained this structure. While wearable cameras such as those used in this paper are an objective means of recording the holistic food and beverage consumption process, their widescale use in the general population is not feasible. Signal-contingent EMA delivered on a personalized schedule to capture near real-time dietary and contextual data or event-contingent EMA triggered by the detection of eating-related behaviors via wearable sensors are some methods that may be appropriate for capturing dietary data with high variability. 

## Figures and Tables

**Table 1:** Cut-offs used to categorize meal timing variability metric scores as low, moderate, high, and very high variability and the equivalent difference in number of hours or eating occasions (EOs) over three days.

	CPD (First and Last EO)		CV No. of EOs		CV Eating Window	
	Cut-off	Hours	Cut-off	EOs	Cut-off	Hours
Variability						
Low	<1.15	<2 h	<10%	<1 EO	<10%	<2 h
Moderate	1.15–1.70	2–3 h	11–20%	1–2 EOs	11–20%	2–4 h
High	1.71–2.40	3–4 h	21–30%	2–3 EOs	21–30%	4–5 h
Very high	>2.40	> 4 h	>30%	>3 EOs	>30%	>5 h

**Table 2:** Participant characteristics and differences between male and female participants.

	All Participants (*n* = 41)	Female (*n* = 27)	Male (*n* = 14)
Age (years)			
18–24	25	18	7
25–30	16	9	7
**Body Mass Index (BMI, kg/m^2^)**			
<18.5	0	0	0
≥18.5 < 25	28	20	8
≥25 < 30	7	3	4
≥30	6	4	2
**Socio-economic status (SES)**			
Higher	22	13	9
Lower	19	14	5
**Highest education attained**			
Secondary school or less	15	10	5
Trade or diploma	7	7	0
University degree	19	10	9
**Employment/study**			
Full-time study	28	18	10
Full-time work	5	4	1
Part-time study/work	5	3	2
Not studying or working	3	2	1

**Table 3:** Mean and range of eating pattern and intra-individual variability metrics.

	All Days (*n* = 123)		
	Mean	Min	Max
Eating pattern metrics			
Time of first EO (hh:mm)	10:18	00:46	19:52
Time of last EO (hh:mm)	20:06	12:43	23:38
No. of EOs per day	4.7	1.0	9.0
Daily eating window (h)	9.8	0.3	22.2
Daily energy intake (kJ)	8478	760	22,879
Intra-individual variability metrics			
CPD First (h)	2.9	0.3	16.3
CPD Last (h)	1.8	0.2	5.8
CV No. of EOs (%)	28.3	0.0	78.1
CV Eating Window (%)	25.6	1.6	106.0

EO, eating occasion; CPD, Composite Phase Deviation; CV, coefficient of variation. CPD First: CPD (i.e., average deviation in hours from a perfectly regular pattern of meal timing) of the first EO for each participant over the three days of data collection. CPD Last: CPD of the last EO for each participant over the three days of data collection. CV No. of EOs: CV for the total daily number of EOs for each participant over the three days of data collection. CV Eating Window: CV for the daily eating window (the duration of time between the first and last EOs of the same calendar day) for each participant over the three days of data collection.

**Table 4:** Associations between eating pattern metrics and Body Mass Index (BMI, kg/m^2^).

	Mean ± SD		*p*-Value
Body Mass Index (BMI)	<25 kg/m^2^ (*n* = 28)	≥25 kg/m^2^ (*n* = 13)	
Time of first EO (hh:mm)	10.58 ± 2.114	9.777 ± 1.021	0.338
Time of last EO (hh:mm)	20.00 ± 1.428	20.41 ± 1.116	0.589
No. of EOs per day	4.726 ± 1.247	4.615 ± 0.989	0.709
Daily eating window (h)	9.419 ± 2.463	10.63 ± 1.423	0.195
Daily energy intake (kJ)	8169 ± 2258	9143 ± 2487	0.311

**Table 5:** Associations between day-to-day variability and demographic variables.

	Mean ± SD		*p*-Value
Gender	Male (*n* = 14)	Female (*n* = 27)	
CPD First (h)	4.128 ± 4.661	2.295 ± 1.772	0.176
CPD Last (h)	1.555 ± 0.805	2.009 ± 1.375	0.263
CV No. of EOs (%)	22.96 ± 9.909	31.00 ± 17.538	0.122
CV Eating Window (%)	24.82 ± 22.59	25.98 ± 24.43	0.883
Body Mass Index (BMI)	<25 kg/m^2^ (*n* = 28)	≥25 kg/m^2^ (*n* = 13)	
CPD First (h)	2.466 ± 1.784	3.903 ± 4.922	0.325
CPD Last (h)	1.843 ± 1.310	1.879 ± 1.045	0.932
CV No. of EOs (%)	30.54 ± 16.75	23.32 ± 12.36	0.174
CV Eating Window (%)	24.68 ± 24.87	27.53 ± 21.19	0.723
Socioeconomic Status (SES)	Top five deciles (*n* = 22)	Bottom five deciles (*n* = 19)	
CPD First (h)	3.786 ± 4.012	1.920 ± 1.091	0.047 *
CPD Last (h)	1.890 ± 1.327	1.813 ± 1.115	0.843
CV No. of EOs (%)	27.58 ± 14.97	29.03 ± 16.90	0.773
CV Eating Window (%)	28.07 ± 24.86	22.70 ± 22.23	0.474

* Significance at *p* ≤ 0.05

## Data Availability

The data presented in this study are available on request from the corresponding author subject to ethical approval.

## Supplementary Materials

The following supporting information can be downloaded at: https://www.mdpi.com/article/10.3390/nu14204349/s1, Table S1: Mean and range of eating pattern metrics across weekdays and weekends; Table S2: Associations between day-to-day variability and total energy intake (kJ) across three days.
