# Supplementary material for: Wearable Cameras Reveal Large Intra-Individual Variability in Timing of Eating among Young Adults
Source: Nutrients. 2022 Oct 17;14(20):4349. doi: 10.3390/nu14204349 (PMC9611808; doi:10.3390/nu14204349)
Supplement: Supplementary file 1 [file nutrients-14-04349-s001.zip › nutrients-1952654-supplementary.pdf]

Supplementary Material

Table S1. Mean and range of eating pattern metrics across weekdays and weekends.

	Weekdays (n = 88)			Weekends (n = 35)		
**Eating Pattern Metrics**	**Mean**	**Min**	**Max**	**Mean**	**Min**	**Max**
Time of first EO (hh:mm)	10:00	00:46	19:52	11:07	01:12	14:11
Time of last EO (hh:mm)	20:10	16:56	23:38	20:04	12:43	23:10
No. of EOs per day	4.7	2.0	9.0	4.6	1.0	9.0
Daily eating window (h)	10.1	1.0	22.2	8.9	0.3	21.4
Daily energy intake (kJ)	8,320	982	17,242	8,875	760	22,879

EO, eating occasion

Table S2. Associations between day-to-day variability and total energy intake (kJ) across three days.

Meal Timing Stability Metric	Correlation Coefficient	p-value
CPD First (h)	0.195	0.222
CPD Last (h)	-0.231	0.146
CV No. of EOs (%)	-0.197	0.218
CV Eating Window (%)	-0.092	0.567

EO, eating occasion; CPD, Composite Phase Deviation; CV, coefficient of variation'

CPD First: CPD (i.e., average deviation in hours from a perfectly regular pattern of meal timing) of the first EO for each participant over the three days of data collection

CPD Last: CPD of the last EO for each participant over the three days of data collection

CV No. of EOs: CV for the total daily number of EOs for each participant over the three days of data collection

CV Eating Window: CV for the daily eating window (the duration of time between the first and last EOs of the same calendar day) for each participant over the three days of data collection
